# Clinical profile, management and outcomes of patients with pulmonary embolism: a retrospective tertiary centre study in Angola

**DOI:** 10.5830/CVJA-2017-017

**Published:** 2017

**Authors:** Ana Manuel, Tomáz Peralta, Abel Salas, José Ricardo, Pedro Sabola, Domingas Baião, Telmo Martins, António Pedro Filipe Júnior, Adelina Aufico, Rui Africano, Adelaide Silva, Carlos Sotolongo, Carlos Sotolongo, António Dias Neto, Vasco Sabino, Joaquim van Dúnem

**Affiliations:** Cardiothoracic Center, Girassol Clinic, Luanda, Angola; Cardiothoracic Center, Girassol Clinic, Luanda, Angola; Cardiothoracic Center, Girassol Clinic, Luanda, Angola; Cardiothoracic Center, Girassol Clinic, Luanda, Angola; Cardiothoracic Center, Girassol Clinic, Luanda, Angola; Cardiothoracic Center, Girassol Clinic, Luanda, Angola; Cardiothoracic Center, Girassol Clinic, Luanda, Angola; Cardiothoracic Center, Girassol Clinic, Luanda, Angola; Intensive Care Unit, Girassol Clinic, Luanda, Angola; Intensive Care Unit, Girassol Clinic, Luanda, Angola; Intensive Care Unit, Girassol Clinic, Luanda, Angola; Intensive Care Unit, Girassol Clinic, Luanda, Angola; Intensive Care Unit, Girassol Clinic, Luanda, Angola; Intensive Care Unit, Girassol Clinic, Luanda, Angola; Imaging Department, Girassol Clinic, Luanda, Angola; Studies Office, Girassol Clinic, Luanda, Angola

**Keywords:** pulmonary embolism, pulmonary CT angiography, anticoagulation, cardiovascular disease, Angola, Africa

## Abstract

**Objective:**

Pulmonary embolism (PE) is a potentially fatal disease. In Angola, few data are available on its occurrence. The aim of the study was to characterise the clinical profile, management and outcomes of patients with PE.

**Methods:**

A retrospective observational study was conducted at the Girassol Clinic in Luanda, Angola. The medical records of patients admitted to the intensive care unit were analysed from 2011 to 2015.

**Results:**

Fifty patients were included and the median age was 50.5 ± 17.8 years. Dyspnoea and immobilisation for more than 72 hours were the most frequently seen risk factors at admission; 28% of the patients had massive PE, 36% sub-massive PE, 28% were haemodynamically unstable at admission and 30% had a very high risk of mortality. The in-hospital mortality rate was 20%.

**Conclusions:**

The clinical characteristics of our patients were similar to those described in the literature. The high prevalence of patients with very high risk at admisson highlights the need to investigate the cause of worst cardiovascular disease outcomes in Africans.

## Introduction

Pulmonary embolism (PE) is characterised by obstruction of the pulmonary arteries by thrombus. It is a potentially fatal disease in the absence of timely diagnosis and treatment. Venous thromboembolism is the third most frequent cardiovascular disease in some Western countries.^1^,^2^ In Africa, the available data relate to retrospective in-hospital and cohort studies, describing the mortality rate between 9.2 and 64%.^3^-^6^

The Virchow triad describes the main factors associated with thrombus formation: blood stasis, hypercoagulable states and endothelial injury. Despite sharing risk factors, deep-vein thrombosis is three times more frequent than PE, and both diseases can co-exist or occur alone.^1^,^7^,^8^

In Angola, few data are available on the occurrence and treatment of PE. Considering the need to improve knowledge about cardiovascular diseases, this study presents the clinical profile, management and outcomes of patients with PE.

## Methods

A retrospective, single-centre, observational study was conducted at the Girassol Clinic in Luanda, Angola. The study was approved by the clinical studies ethics committee of the Girassol clinic polyvalent intensive care unit (ICU). The manuscript is in accordance with the Helsinki Declaration and with ethical guidelines from our studies committee.

The medical records of patients admitted to our ICU were analysed from September 2011 to September 2015. Clinical suspicion was defined by the physician based on symptoms, signs and risk factors. Patients with clinical suspicion were included in the study if PE was confirmed by one of the following diagnostic tests: pulmonary computed tomography (CT) angiography, transthoracic echocardiography and Doppler ultrasound of the limbs.

**Fig. 1:**
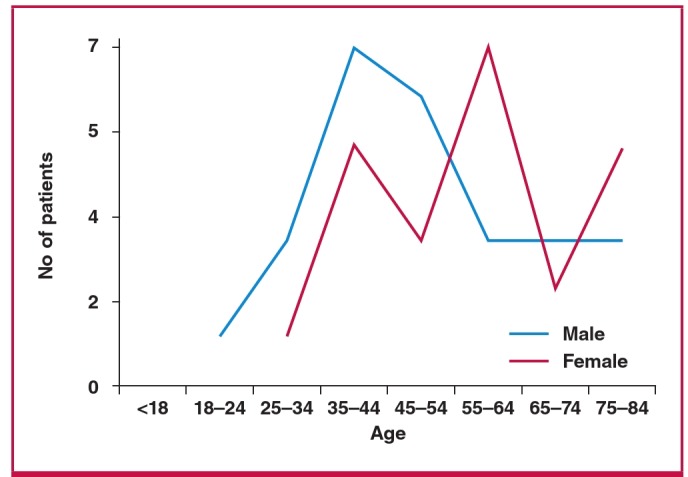
Age and gender of the patients with pulmonary embolism.

Demographic variables and the presence of symptoms and clinical signs of PE were analysed (Fig. 1, Table 1). The presence of risk factors and co-morbidities is described in Table 2. Patients were also stratified according to PE risk scores (modified Wells and Geneva revised scoring systems).

**Table 1:** Prevalence of symptoms and signs of patients with pulmonary embolism at admission

*Symptoms and signs*	*Number (%)*
Dyspnoea	34 (68)
Chest pain	20 (40)
Cough	9 (18)
Lower-limb pain	7 (14)
Tachycardia	6 (12)
Altered consciousness	5 (10)
Anxiety	3 (6)
Cyanosis	1 (2)
Syncope	1 (2)
Cardiorespiratory arrest	1 (2)
Other symptoms	15 (30)
Asymptomatic	2 (4)

**Table 2:** Risk factors and co-morbidities of patients with pulmonary embolism

*Risk factors and co-morbidities*	*PE, n (%)*
Immobilisation > 72 hours	24 (48)
Hospitalisation/surgery < 3 months	14 (28)
Arterial hypertension	18 (36)
Recent trauma	8 (16)
Diabetes mellitus	6 (12)
Obesity	6 (12)
Cancer	5 (10)
Previous known coagulations disorders	4 (8)
Smoking	4 (8)
Coronary artery disease/previous AMI	3 (6)
Hormonal treatment	3 (6)
Deep-vein thrombosis	3 (6)
Dyslipidaemia	2 (4)
Heart failure	2 (4)
COPD	1 (2)
Previous PE < 3 months	1 (2)
Stroke	1 (2)
Sickle cell disease	1 (2)
Pregnancy	1 (2)
Central venous catheter	1 (2)
Atrial fibrilation	1 (2)
Chronic kidney disease	1 (2)
No risk factors or co-morbidities	3 (6)

AMI, acute myocardial infarction; COPD, chronic obstructive pulmonary disease.

The following diagnostic tests were analysed regarding the frequency of realisation and positivity rates:

Laboratory tests: D-dimer, troponins, B-type natriuretic peptide (BNP), increased white blood cell count, increased erythrocyte sedimentation rate and lactate dehydrogenase enzyme (LDH) levels.
Arterial gasometry: presence of hypoxaemia, acute respiratory alkalosis and changes not related to PE.
Chest X-ray: presence of atelectasis, parenchymal infiltrates, pleural effusion, pneumothorax, cardiomegaly, Westmark and Hampton signs and changes not related to PE.
ECG: presence of sinus tachycardia, S1Q3T3 pattern, pulmonary P wave, right bundle branch block, right ventricular hypertrophy, right cardiac axis deviation, reversal of T wave in V1–V3 leads and unspecific alterations of repolarisation.
Echocardiogram: presence of enlargement or thrombus in the right chambers, right ventricle (RV) hypokinesia, McConnell sign, persistent pulmonary hypertension, patent foramen ovale and changes not related to PE.
Doppler ultrasound of limbs: presence of thrombi or decreased venous compressibility.
Pulmonary computed tomography angiography (PCTA): the lesions were classified as massive PE if the thrombosis was in a central location (main and lobar branches); patients with thrombosis in the segmental and sub-segmental branches were classified as sub-massive PE if RV dysfunction was present; and they were classified as low-risk PE on the absence of thrombus.

Patients were also classified as haemodynamically unstable if their systolic blood pressure was under 90 mmHg, or there was poor peripheral perfusion or cardiogenic shock, and according to the pulmonary embolism severity index (PESI).^9^

The treatment type and duration was analysed. The following complications were considered: death; reversed cardiorespiratory arrest; heart failure; respiratory failure requiring mechanical ventilation; major bleeding, cardiogenic shock, acute myocardial infarction, acute kidney injury (AKI) or chronic kidney disease agudisation, sepsis originating in the respiratory tract, hyperglycaemia > 200 mg/dl (11.1 mmol/l) in non-diabetic patients, and peripheral embolisation.

Data are presented using tables with absolute and relative frequencies, average arithmetic values and standard deviations. Statistical analysis was performed as two-sided significance tests. The non-parametric chi-squared test was used to test heterogeneity of proportions.

## Results

A total of 50 patients were included and the median age was 50.5 ± 17.8 years. The age groups 35 to 44 years and 55 to 64 years were the most affected (Fig. 1), 72% of patients were over the age of 40 years, 52% were male and 86% were black.

Respiratory symptoms, including dyspnoea (68%), chest pain (40%) and cough (18%) were the most frequent. Only 4% of patients were asymptomatic and one patient presented with cardiorespiratory arrest (Table 1).

Risk factors and more prevalent co-morbidities were immobilisation for more than 72 hours (48%), hospitalisation or recent surgery (28%), and hypertension (36%). In three patients (6%) there were no identified risk factors or co-morbidities (Table 2).

The estimated pre-test probability of PE was analysed according to the Wells and Geneva criteria. Fifty-six and 58% of patients had moderate and intermediate PE probability, respectively (Table 3).

**Table 3:** Pulmonary embolism risk stratification according to the modified Wells and Geneva revised scoring systems

*PE probability*	*Wells scoring system n (%)*	*Geneva scoring system n (%)*
Low	7 (14)	5 (10)
Moderate	28 (56)	
Intermediate		29 (58)
High	15 (30)	16 (32)
Total	50	50

Laboratory tests deviated from normal in 68% of patients; their positivity rates and cut-off values are shown in Table 4. D-dimer, troponin and BNP levels were positive in all tests with available results. More than half of the patients (54%) had abnormal results in their arterial blood gasometry (ABG), namely hypoxaemia in 34% and acute respiratory alkalosis in 20% of patients.

**Table 4:** 

*Diagnostic tests*	*Number (%)*
Laboratory tests	
White blood cells (> 10 × 10^9^ cells/l)	14 (28)
LDH (> 400 U/l)	10 (20)
D-dimers (> 500 μg/l)	4 (8)
Troponins (> 0.1 ng/ml)	4 (8)
ESR (> 10 mm in men, > 20 mm in women)	3 (6)
BNP (> 500 pg/ml)	1 (2)
Normal	16 (32)
Arterial blood gasometry (ABG)	
Hypoxaemia	17 (34)
Acute respiratory alkalosis	10 (20)
Normal	13 (26)
Absent ABG	13 (26)
Chest radiography	
Pulmonary parenchymal infiltrates	7 (14)
Hampton sign	6 (12)
Pleural effusion	4 (8)
Cardiomegaly	2 (4)
Pneumothorax	1 (2)
Westmark sign	1 (2)
Changes not related to PE	5 (10)
Normal	13 (26)
Absent chest radiography	12 (24)
Electrocardiogram	
S1Q3T3 pattern	9 (18)
Non-specific repolarisation changes	6 (12)
Right bundle branch block	4 (8)
Right ventricular hypertrophy	1 (2)
Right cardiac axis deviation	1 (2)
Sinus tachycardia	1 (2)
Changes not related to PE	6 (12)
Normal	11 (22)
Absent ECG	11 (22)
Echocardiogram	
Enlarged right heart chambers with or without thrombus	10 (20)
Right ventricular hypokinesis	4 (8)
Pulmonary hypertension	3 (6)
Persistent foramen ovale	2 (4)
McConnel sign	2 (4)
Changes not related to PE	4 (8)
Normal	9 (18)
Absent echocardiogram	17 (34)
Limb Doppler ultrasound	
Deep-vein thrombosis	10 (20)
Normal	33 (66)
Absent Doppler ultrasound	7 (14)

LDH, lactate dehydrogenase enzyme; ESR, erythrocyte sedimentation rate; BNP, B-type natriuretic peptide.

About a quarter of patients (13 patients) had normal chest radiography. The electrocardiogram was normal in only 22% of patients and the classic S1Q3T3 pattern was found in only 18%. RV enlargement (20%) and RV hypokinesia (8%) were the main echocardiographic findings in our study. We documented the presence of deep-vein thrombosis by Doppler ultrasound in 20% of patients.

PCTA changes were correlated with haemodynamic stability at admission in all patients; 28% had massive PE, of whom 20% were haemodynamically unstable; 36% had sub-massive PE and showed a statistically significant rate of haemodynamic stability at admission (28 vs 8%, p = 0.018). In 36% of patients there was low-risk PE (Table 5). All patients were stratified according to the pulmonary embolism severity index and 30% of the patients had a very high risk of mortality (Table 6).

**Table 5:** Pulmonary embolism classification according to pulmonary computed tomography angiography and correlation with haemodynamic stability at admission

*CT angiography classification*	*Haemodynamically stable patients, n (%)*	*Haemodynamically unstable patients, n (%)*	*Sub-total*	*p-value*
Massive PE	4 (8)	10 (20)	14 (28)	0.109
Sub-massive PE	14 (28)	4 (8)	18 (36)	0.018
Low-risk PE	18 (36)	-	18 (36)	-
Total	36 (72)	14 (28)	50	

**Table 6:** Stratification of patients according to the pulmonary embolism severity index

*30-day mortality risk classes*	*Number (%)*
I: Very low risk (0–1.6%)	17 (34)
II: Low risk (1.7–3.5%)	10 (20)
III: Moderate risk (3.2–7.1%)	5 (10)
IV: High risk (4.0–11.4%)	3 (6)
V: Very high risk (10.0–24.5%)	15 (30)
Total	50

Heparins were the most common form of in-hospital anticoagulation. Unfractionated heparin was used in 32% of patients for 5.4 ± 2.1 days. Low-molecular-weight heparins were used in 44% of patients for 6.2 ± 3.7 days. Among these patients, 70% used oral anticoagulation with warfarin and 6% used new oral anticoagulants (NOAC) (Table 7). Thrombolytic therapy was used in 18% of the patients. In 12 patients, it was not possible to determine the type of anticoagulant used or whether they used thrombolytic therapy, due to the unavailability of data.

**Table 7:** Treatment of pulmonary embolism

*Treatment*	*Number (%)*	*Median duration (days) ± SD*
Thrombolytic therapy	9 (18)	-
Unfractionated heparin	16 (32)	5.4 ± 2.1
Low-molecular-weight heparins	22 (44)	6.2 ± 3.7
Warfarin	35 (70)	Continuous use after discharge
New oral anticoagulants	3 (6)	Continuous use after discharge
Elastic compression bandage	3 (6)	Continuous use after discharge
Unavaible treatment information	12 (24)	-

There were complications in 38% of the patients; 15 had respiratory failure requiring mechanical ventilation and seven had cardiogenic shock (Table 8). The 24-hour and in-hospital mortality rates were 2.5 and 20%, respectively. There were 15 deaths, of which five occurred in the first 24 hours after admission.

**Table 8:** 

*Complications*	*Massive PE*	*Sub-massive PE*	*Low-risk PE*	*Number (%)*
Respiratory failure requiring mechanical ventilation	12	3	-	15 (37)
Cardiogenic shock	4	3	-	7 (18)
Sepsis and pulmonary infection	2	2	1	5 (13)
Cardiorespiratory arrest (reversed)	3	1	-	4 (10)
AKI or chronic kidney disease agudisation	3	1	-	4 (10)
Acute myocardial infaction	2	-	-	2 (5)
Heart failure	1	1		2 (5)
Hyperglycaemia > 200 mg/dl (11.1 mmol/l) in non-diabetic patients	-	-	1	1(2)
Sub-total	27 (67)	11 (28)	2 (5)	40

AKI, acute kidney injury.

Considering the study criteria, three patients had a fatal outcome and were not included (no imaging confirmation of PE). However, they showed a high clinical probability for PE and alternative diagnoses were less likely.

## Discussion

The study results characterise the clinical profile of patients with PE admitted at our hospital. The presented data refer to the current clinical practice without any interference in medical procedures. The high clinical suspicion associated with the immediate availability of pulmonary CT angiography and other diagnostic tests allowed us to confirm PE cases and exclude other differential diagnoses. This context has added greater consistency to the study results.

According to Tambe and colleagues in Cameroon,^10^ it was found that PE is not a rare disease in sub-Saharan African populations. Institutional unavailability of CT angiography may favour sub-detection of the disease in some geographic areas.^10^

The median age observed in our study was 50.5 ± 17.8 years, similar to that described in the EMPEROR study^11^ (56.5 ± 18.1 years), and lower than some observational studies describing median ages between 60 and 68.9 years.^7^,^12^-^14^ According to Memtsoudis,^15^ in a retrospective and multicentre study of patients with PE after arthroplasty, the regressive multivariate analysis suggested that there is a higher risk of PE in the age group 45 to 64 years, but age alone was not identified consistently as a risk factor.

Black patients were predominant (86.9%) in our study. The fact that the study was conducted in an African country may have contributed to this result. In the EMPEROR study,^11^ conducted in a population with multiple ethnic groups, they found a prevalence of 25.6% Afro-American patients with PE. Evidence suggested that non-Caucasian origin could be predictive of worse clinical outcomes for acute cardiovascular disease.^11^,^16^

The most common symptoms in our study were dyspnoea, chest pain and cough. These results are similar to those found in the JASPER study.^12^ However tachypnoea and tachycardia have been reported at higher prevalences compared to our results.^7^,^14^,^17^

The clinical manifestations of PE are often unspecific, which represents a diagnostic challenge. Dyspnoea and chest pain are symptoms that may constitute the sole or first manifestation of a broad spectrum of diseases. The observation of sudden dyspnoea may suggest PE. However, few studies describe a correlation between the degree of dyspnoea perceived by the patient and the degree observed by physicians.^18^ Chest pain associated with PE may have pleuritic or anginal characteristics in cases of RV ischaemia.^14^

In a cohort study conducted in primary healthcare, the most common differential diagnoses in patients referred for suspected PE were chest pain/non-specific dyspnoea, pneumonia, myalgia, asthma/COPD, hyperventilation anxiety disorders, heart failure, pericarditis and lung cancer. In these patients, although PE was excluded, there was a greater probability of clinically relevant illness in the presence of sudden dyspnoea, tachycardia, cough and haemoptysis.^18^

The most prevalent risk factor in our study was immobilisation for more than 72 hours in 48% of patients. Similar results were found in the ICOPER^7^ and EMEP^14^ studies in 28 and 38.5% of patients, respectively. The effect of the muscle pump in maintaining venous return is considered one of the main promotional mechanisms of blood stasis in immobilised patients.^14^

The prevalence of patients over 40 years of age was 72% in our study. The incidence of venous thromboembolic events increases after 40 years and it is estimated that the risk doubles with each subsequent decade.^19^

The prevalence of patients with cancer (10%) was lower than the range of 24.3 to 18.3% reported in other studies.^7^,^12^,^14^,^20^ This result may have been influenced by the prevalence of cancer in the different populations studied. In a retrospective study in cancer patients, PE was an accidental imaging found in 69.4% of patients. Cancer increases the risk of venous thromboembolism, mainly by activation of the coagulation system. Some authors suggest a systematic investigation for cancer in patients with PE of undetermined aetiology, and the prevention of thrombosis in patients with cancer.^21^

In the RIETE registry,^22^ predictors for PE were found to be increased mortality rate, type of venous thromboembolism, advanced age, cancer and immobilisation due to neurological disease.^22^ In 6% of our patients, no risk factors or co-morbidity were identified. It was recognised that in some patients, aetiology of PE may not be determined, suggesting the existence of unknown risk factors associated with the heterogeneity of individual susceptibility.^23^,^24^

The majority of patients in our study had moderate/intermediate PE probability. Although the Wells score includes subjective criteria, overall accuracy as a clinical prediction rule is similar to the Geneva score, as previously reported.^2^ In our study, the frequency of intermediate and high-probability PE groups was similar for both scoring systems. Of note, only 10 to 14% of the patients had a low probability.

Since requesting D-dimer blood tests is less likely in patients with higher PE probability, there was a low frequency of realisation and positivity rates of D-dimer blood tests. It is notable that D-dimer, troponin and BNP tests were positive in all our patients with available results. However, there were no D-dimers and cardiac biomarkers measured in more than 50% of our patients, a higher percentage than that found in the SWIVTER register,^25^ in which 30% of patients with PE had no cardiac biomarkers or echocardiograms done.^25^ This result may reflect under-utilisation of these tests, or it may just be unavailability of records of the laboratory tests performed.

The ABG, chest radiography, electrocardiogram, echocardiogram and Doppler ultrasound of the limbs were performed in more than 75% of our patients. In 54% of these patients, there were deviations from normal in the ABG, and the most frequent was hypoxaemia. Bova et al. described hypoxaemia as an independent predictor of PE mortality at three months.^26^

Abnormal chest radiography was documented in 50% of our patients. These results are similar to those found in the EMEP study (45.8%).^14^ According to these authors, interobserver variability and subjectivity in the interpretation of chest radiography may have influenced the results.

Electrocardiographic (ECG) changes were identified in 56% of patients; however, isolated use of ECG has low sensitivity and specificity to exclude PE. ECG is an important test for the evaluation of diseases with similar presentation to that of PE in the acute phase and can be included in some risk-stratification strategies.^27^

In the 48% of patients with abnormalities on the echocardiogram, changes in the right chambers were the most frequent. This examination is often used in the evaluation of patients with PE and has the advantage of not being invasive or expensive. Although there is inter-observer variability and different evaluation criteria, the echocardiogram allows identification of RV dysfunction, which is described as one of the main predictors of early mortality in patients with sub-massive PE.

It is estimated that about 30 to 40% of normotensive PE patients at admission have RV dysfunction, identifiable by echocardiography. These patients have in-hospital mortality rates between 11.8 and 23%, substantially higher than the rates of normotensive patients without RV dysfunction (0–9.6%).^28^

Lower-limb Doppler ultrasound in symptomatic patients with deep-vein thrombosis (DVT) has a sensitivity of 96% and specificity of 99%. It is an important examination considering that about 70% of patients with PE have lower-limb DVT.^29^

In our study, 20% of the patients presented with DVT. The inter-observer variability and limitations of ultrasound in identifying thrombi in the pelvis and in small vessels of the leg may have influenced this result.

PCTA is the imaging test of choice for diagnosis and exclusion of PE, considering its high sensitivity and specificity.^30^ The correlation of its changes with haemodynamic stability at admission showed that most of our patients with massive PE had haemodynamic instability, as previously described.^31^ Additionally, we found a statistically significant rate of haemodynamic stability at admission for patients with sub-massive PE.

A meta-analysis to assess the prognostic value of the embolic load for short-term mortality showed that the presence of emboli centrally located in a pulmonary artery was associated with twice the risk of mortality within 30 days.^32^ On the other hand, assessment of RV function by pulmonary CT angiography has diagnostic and prognostic value in PE.^30^,^33^

In this study, 28% of patients presented with sub-massive PE, despite haemodynamic stability at admission. The presence of RV dysfunction and centrally located pulmonary artery thrombus predicts a higher mortality rate in normotensive patients.^30^ Low-risk 30-day-mortality PE was identified in 36% of patients and all were stable at admission. Studies show that these patients have a lower risk of adverse events and may be candidates for home treatment.^34^,^35^

The treatment of PE in the acute phase, based on anticoagulation with heparin or NOAC, prevents the extension of thrombi and recurrence of thromboembolic events. We found that most of our patients received low-molecular-weight heparins and warfarin. Out-patient anticoagulation depends on the clinical context and existing risk factors, and its duration in PE is still controversial.

In our study, thrombolytic therapy was used in 18% of patients, however 28% presented with haemodynamic instability. These results may suggest an underuse of thrombolytic therapy. Similar results were found in the EMEP study^14^ in which 20% of the patients were hypotensive but thrombolytic therapy was used in only 15% of them. Thrombolysis allows early pulmonary reperfusion, and despite increasing the risk of major bleeding, is indicated in unstable patients. Furthermore, it benefits normotensive patients with sub-massive PE, preventing haemodynamic instability, as demonstrated in the PEITHO study.^35^

The in-hospital mortality rate of 20% in our study was similar to that described in the EMEP study (22%) and higher than the rate described in other PE studies.^17^,^14^,^22^,^36^ Conducting the study in the ICU on more severely ill patients with a rate of 38% complications may have contributed to the higher mortality rate. Moreover, we found that 67% of the complicating events occurred in the PCTA sub-group of massive PE; 30% of patients had a very high risk of 30-day mortality, according to the admission PESI score; and a third of the deaths occurred within the first 24 hours of hospitalisation, which may reflect the severity of PE since admission.

Some limitations of this study relate to its retrospective nature and the lack of data. In addition, using health professionals from different schools may have favoured some variability in clinical practice during the study period.

## Conclsuions

Our results confirm that PE does not seem to be a rare disease in African populations. The clinical characteristics of the study sample were similar to those described in the literature, although black patients were more prevalent. In diagnostic examinations, the use of pulmonary CT angiography in all patients allowed consistent diagnosis and assessment of the prognosis. Most patients were treated with low-molecular-weight heparin and warfarin. The intra-hospital mortality rate was relatively higher than that described in other studies. The high prevalence of patients with very high risk of mortality at admission highlights the need to investigate the cause of worst cardiovascular disease outcomes in Africans.
